# Noncanonical necrosis in 2 different cell types in a *Caenorhabditis elegans* NAD^+^ salvage pathway mutant

**DOI:** 10.1093/g3journal/jkac033

**Published:** 2022-02-10

**Authors:** Rifath N Reza, Nicholas D Serra, Ariana C Detwiler, Wendy Hanna-Rose, Matt Crook

**Affiliations:** 1 Department of Biochemistry and Molecular Biology, The Pennsylvania State University, University Park, PA 16802, USA; 2 Department of Genetics, University of Pennsylvania, Philadelphia, PA 19104, USA; 3 Department of Environmental and Occupational Health, University of Pittsburgh Graduate School of Public Health, Pittsburgh, PA 15261, USA; 4 Department of Life Sciences, Texas A&M University-San Antonio, San Antonio, TX 78224, USA

**Keywords:** necrosis, NAD^+^ salvage, nicotinamide, calcium, aspartic proteases, autophagy, apoptosis, *Caenorhabditis elegans*

## Abstract

Necrosis was once described as a chaotic unregulated response to cellular insult. We now know that necrosis is controlled by multiple pathways in response to many different cellular conditions. In our *pnc-1* NAD^+^ salvage deficient *Caenorhabditis elegans* model excess nicotinamide induces excitotoxic death in uterine-vulval uv1 cells and OLQ mechanosensory neurons. We sought to characterize necrosis in our *pnc-1* model in the context of well-characterized necrosis, apoptosis, and autophagy pathways in *C. elegans*. We confirmed that calpain and aspartic proteases were required for uv1 necrosis, but changes in intracellular calcium levels and autophagy were not, suggesting that uv1 necrosis occurs by a pathway that diverges from *mec-4d*-induced touch cell necrosis downstream of effector aspartic proteases. OLQ necrosis does not require changes in intracellular calcium, the function of calpain or aspartic proteases, or autophagy. Instead, OLQ survival requires the function of calreticulin and calnexin, pro-apoptotic *ced-4* (Apaf1), and genes involved in both autophagy and axon guidance. In addition, the partially OLQ-dependent gentle nose touch response decreased significantly in *pnc-1* animals on poor quality food, further suggesting that uv1 and OLQ necrosis differ downstream of their common trigger. Together these results show that, although phenotypically very similar, uv1, OLQ, and touch cell necrosis are very different at the molecular level.

## Introduction 

Necrosis was once thought to be a chaotic form of cell death caused by external insults, compared with the precise control of apoptosis in response to intrinsic signals. A wide body of research over the last 30 years has shown that necrosis of different cell types in different species share common characteristics and mechanisms (McCall 2010). As the study of cell death in general, and necrosis in particular, has expanded so have the different categories of cell death, depending on insult, mechanism, and characteristics (Fricker *et al.* 2018). The initiators of necrosis are many and varied, including the inhibition of apoptosis and DNA damage, increased glutamate levels, inflammation, ischemia, reactive oxygen species, and cellular calcium levels (Fricker *et al.* 2018). Necrosis can occur in a range of tissues but has been especially well studied in the context of neurodegeneration (Mattson 2007), ischemic reperfusion injury (Schinzel *et al.* 2005), muscular dystrophy (Millay *et al.* 2008), and heart disease (Kung *et al.* 2011). It is typically defined by its physiological characteristics compared with apoptosis. These include plasma membrane whorls, vacuole formation, cytoplasmic swelling, and nuclear membrane disintegration with a lack of chromatin condensation (Hall *et al.* 1997). Biochemical characteristics of necrosis often include depletion of ATP (Laporte *et al.* 2007), opening of the mitochondrial permeability transition pore (mPTP) (Kinnally *et al.* 2011), increase in NAD^+^ catabolism by poly[ADP-ribose] polymerases (PARPs) (Fatokun *et al.* 2014), over production of glutamate and N-methyl D-aspartic acid (NMDA) (Lai *et al.* 2014) and a rapid increase in intracellular calcium (Choi 1987).

Necrosis can also be defined by the mechanisms and pathways that execute the necrotic death program. Common to many types of necrosis, such as oncosis, lysosomal necrosis, and mitopore necrosis, is an increase in intracellular calcium (Fricker *et al.* 2018). This increase can be the result of plasma membrane dysfunction leading to an increase in Na^+^/Ca^2+^ pumping (Bano *et al.* 2005) or calcium release from the endoplasmic reticulum (ER) (Xu *et al.* 2001; Bianchi *et al.* 2004) or mitochondria (Lemasters *et al.* 2009; Rasola and Bernardi 2011). Increasing intracellular calcium levels activate calpain proteases and cathepsins, dubbed the calpain-cathepsin hypothesis (Yamashima 2004). An expansion of lysosomes (Artal-Sanz *et al.* 2006) and increased vacuolar H^+^-ATPase mediated acidification (Syntichaki *et al.* 2005) leads to lysosome rupture with the activation of aspartic proteases and the disintegration of the plasma membrane. *Caenorhabditis* *elegans* touch cell necrosis in *mec-4*, *deg-1*, or *Gαs* gain-of-function backgrounds has been an excellent model to study the mechanisms of necrosis (Driscoll and Chalfie 1991). *mec-4* encodes a plasma membrane localized degenerin/epithelial sodium channel (DEG/EnaC), and a channel pore Ala 442 substitution results in the conduction of calcium ions into the cell, inducing the release of ER Ca^2+^ and progression toward necrosis of the posterior and anterior ventral (PVM, AVM) and posterior and anterior lateral (PLM and ALM pairs) touch neurons (Driscoll and Chalfie 1991; Bianchi *et al.* 2004). Mutations in the ER calcium-binding proteins calreticulin and calnexin suppressed touch cell necrosis induced by *mec-4d* and other degenerin ion channel mutations, as did the reduction of cytoplasmic calcium levels by EGTA chelation or the addition of dantrolene, an agonist of the ER membrane ryanodine receptor (Xu *et al.* 2001). Blocking sarco/ER Ca^2+^-ATPase (SERCA) based calcium reuptake into the ER by thapsigarin reversed this suppression (Xu *et al.* 2001). Reducing intracellular calcium levels similarly reduced *clhm-1* overexpression-induced touch cell necrosis (Tanis *et al.* 2013) and a wave of elevated calcium levels from the anterior to the posterior intestine results in the necrosis of the intestine at the onset of death in *C. elegans* (Coburn *et al.* 2013). Support for the calpain-cathepsin hypothesis was provided by the reduction of touch cell necrosis after RNAi knockdown of *clp-1* and *tra-3* (calpain proteases) and *asp-3* and *asp-4* (aspartic proteases) (Syntichaki *et al.* 2002). Functional autophagy is also required for touch cell necrosis as loss or reduction-of-function mutations in the autophagy master regulator *unc-51* and vesicle inducer *bec-1* or RNAi knockdown of *lgg-1* (involved in vesicle elongation) reduces degenerin-induced touch cell necrosis (Toth *et al.* 2007; Samara *et al.* 2008). Supporting the role of autophagy in necrosis, Tor signaling is protective against necrosis and starvation exacerbates ion channel death (Toth *et al.* 2007).

In *C. elegans*, the nicotinamidase PNC-1 catalyzes the first step in the recycling of nicotinamide (NAM) back into nicotinamide adenine dinucleotide (NAD^+^) (Huang and Hanna-Rose 2006; van der Horst *et al.* 2007). uv1 and OLQ cells die in a *pnc-1* mutant due to excess NAM, which overactivates the OCR-4/OSM-9 transient receptor potential vanilloid (TRPV) ion channel (Huang and Hanna-Rose 2006; Vrablik *et al.* 2009; Upadhyay *et al.* 2016). uv1 cells are neuroendocrine cells in the uterus that are thought to play a role in the control of egg laying (Zahn *et al.* 2001; Alkema *et al.* 2005; Jose *et al.* 2007), though this was not supported by later work (Johnson *et al.* 2009). uv1 cells die shortly after specification in the mid-L4 stage, with the classic cellular vacuolization and swelling characteristic of necrotic cells (Huang and Hanna-Rose 2006). The consequences of uv1 necrosis in *pnc-1* animals is unclear, as the *pnc-1* egg-laying phenotype is due to impaired vulval muscle function (Vrablik *et al.* 2011). OLQ cells are mechanosensory neurons responsible in part for the gentle nose touch response, where animals will stop on contact with an object and reverse direction (Kaplan and Horvitz 1993). OLQ cells die in a stereotyped degeneration from early L3 to early L4, beginning with dendrite blebbing, progressing to cytoplasmic swelling and vacuolization and ending with disappeared neurites (Upadhyay *et al.* 2016), much like degenerating dopaminergic neurons after 1-methyl-4-phenylpyridinium (MPP^+^) or 6-hydroxydopamine (6-OHDA) insult or in a dominant *trp-4* channel background (Pu and Le 2008; Nagarajan *et al.* 2014; Offenburger *et al.* 2018). OLQ death increases head bend frequency on nose touch but decreases stop and reverse behavior (Upadhyay *et al.* 2016). uv1 necrosis requires calpain and aspartic proteases (Huang and Hanna-Rose 2006), but neither uv1 nor OLQ necrosis requires the apoptosis regulator CED-4 (Huang and Hanna-Rose 2006; Upadhyay *et al.* 2016) and OLQ survival is significantly worse in a *ced-4(n1163); pnc-1(pk9605)* mutant (Upadhyay *et al.* 2016).

We sought to determine if uv1 and OLQ necrosis are controlled by the same genes and pathways as touch cell necrosis and explore further any potential role for pro-apoptosis and autophagy genes. Our aim is to fit NAM-induced necrosis into the broader picture of necrosis and cell death. We found that while uv1 necrosis shares some molecular characteristics with touch cell necrosis, uv1, and OLQ necrosis are distinct both from each other and touch cell necrosis, with a role for calreticulin, *ced-4*, and axon guidance genes in OLQ survival. Our findings further demonstrate the value of *C. elegans* as a model for studying necrosis and increasing our understanding of necrotic cell death in humans.

## Materials and methods

### Strains and maintenance

Strains were maintained under standard conditions at 20° (Brenner 1974). We used the following alleles:

I *dlk-1(ju476), unc-14(e57)*, tdIs5 (*Pmec-4::gfp*); II *rrf-4(pk1426)*, inIs179 *(ida-1::GFP)*; III *glr-1(n2461), ced-9(n1950), ced-4(n1162) dpy-17(e164), cnx-1(nr2009)*; IV *pnc-1(pk9605)*, *ced-3(n717) unc-26(e205)*; V *egl-1(n1084n3082), unc-51(e369), unc-51(e1189), crt-1(bz29)*; X *asp-4(ok2693), mec-4d(u231)*, *mec-4d(e1611).*

### RNAi

RNAi was carried out by placing 5 L4 animals on 1 mM IPTG 25 µl/ml carbenicillin NGM plates seeded with HT115 bacteria containing the RNAi clone of interest (Ahringer 2006). The offspring of these animals were then scored for OLQ and/or uv1 survival at the late L4 stage.

### Pharmacology

Plates containing 1 mM NAD^+^ (Sigma-Aldrich, St. Louis, MO, USA) or 25 mM NAM (Alfa Aesar, Ward Hill, MA, USA) were made by adding 250 µl of the appropriate concentration stock NAD^+^ or NAM solution to OP50 seeded NGM plates and allowed to dry overnight.

To investigate the role of calcium in necrosis a chelation experiment was carried out using 50 mM EGTA (Acros Organics, West Chester, PA, USA) plates with 1 mM CaCl_2_ (all assays) and 0.5 mM CaCl_2_ (OLQ survival). A similar approach was to use the ER calcium release blocker dantrolene (Calbiochem, San Diego, CA, USA) at 10 µM in plates with no CaCl_2_. Both EGTA and dantrolene were added to liquid NGM agar before pouring. The role of the calcium reuptake blocker thapsigargin in necrosis (Calbiochem, San Diego, CA, USA) was carried out using 0.5 µM plates prepared by adding 250 µl of a 1 mM stock solution in DMSO to OP50 seeded NGM plates with no CaCl_2_.

NGM plates with UV-killed *E. coli* OP50 were made by seeding NGM plates with a fresh *E. coli* OP50, allowing the bacteria to grow for 3–5 days and then exposing them to 254 nm UV for 10 min. Randomly selected plates had the killed lawn streaked on unseeded LB agar plates to confirm the effectiveness of the UV treatment.

### Phenotype scoring

#### Cell death

For each cell death experiment 5–10 L4 animals were placed on each of 5 experimental plates and their progeny were scored at the appropriate developmental stage. All RNAi and pharmacology assays were repeated in triplicate.

##### uv1 necrosis

uv1 cells die by necrosis in a *pnc-1* background shortly after they are specified in the L4 stage (Huang and Hanna-Rose 2006). By using an integrated terminal uv1 cell GFP marker, *ida-1::gfp* (Zahn *et al.* 2001), we are able to count the healthy GFP positive uv1 cells in mid to late L4 animals and calculate percentage survival out of a maximum of 4 uv1 cells *per* animal. For some strains, uv1 survival was scored using the psEx241 *ocr-4p::gfp::ocr-4 3’UTR* (Upadhyay *et al.* 2016) marker by counting GFP positive uv1 cells with normal triangular shape (Huang and Hanna-Rose 2006) as opposed to the round faint GFP positive dying or dead cells. Percentage survival was calculated as above.

##### OLQ death

we scored OLQ death using either the psEx241 (*pnc-1* background) or psEx279 (*rrf-3* background) *ocr-4p::gfp::ocr-4 3’UTR* marker by counting intact GFP positive dendrites in mid-L4 animals and from this calculated percentage survival out of a maximum of 4 OLQ cells *per* animal.

##### Touch cell necrosis

*Caenorhabditis* *elegans* uses 6 mechanosensory neurons to respond to gentle touch; ALML/R, PLML/R, AVM, and PVM (Chalfie and Sulston 1981). The majority of these cells die by necrosis in a *mec-4d* background (Driscoll and Chalfie 1991). We assayed touch cell necrosis using an integrated *Pmec-4d::gfp* marker (tdIs5) in L4 animals by counting GFP positive touch cells and calculated the number of necrotic touch cells *per* 100 animals as [(maximum 6 touch cells × *n*)—total # GFP positive touch cells] × 100 (Syntichaki *et al.* 2002).

#### Nose touch

L4 stage animals were picked to individual NGM plates seeded with live OP50 *E. coli* for testing. An eyelash was placed in the path of the worm, allowing the worm to collide with it nose first (Kaplan and Horvitz 1993). Animals were scored as responding to touch if they either immediately stopped on contact or stopped and reversed. Each worm was allowed to collide with the eyelash 30 times in 3 sets of 10, with 15 s between each test. Each assay was repeated 4 times for a total sample size of 35–40 animals. Data were expressed as % animals responding to touch relative to wild-type animals.

## Results

### uv1 and OLQ death

**Fig. 1.**
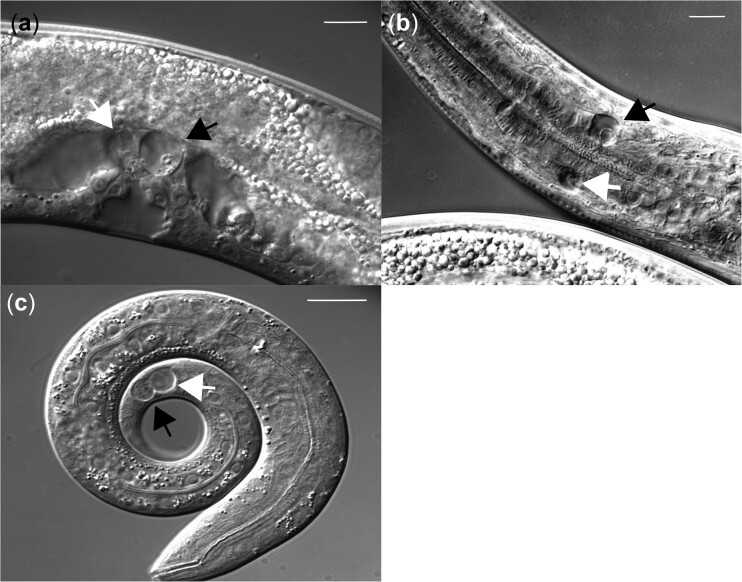
pnc-1 and *mec-4d* mutations result in cell death. In *pnc-1(pk9605)* animals up to 4 uv1 cells (a)(Crook *et al.* 2013) and OLQ mechanosensory neurons (b) die. In a *mec-4d(e1611)* animals up to 6 touch cell neurons die (c), with both dying PLM tail neurons shown. Black and white arrowheads point to dying cells in and out of focal plane, respectively, whereas other dying cells are not visible in the focal planes of the images above. DIC photomicrographs were captured using a Zeiss Axioplan 2 DIC microscope. Scale bar is 10 µm.

In *pnc-1(pk9605)* animals dying uv1 (Fig. 1a) and OLQ cells (Fig. 1b) show the same characteristic necrotic phenotype of vacuolar swelling and break down of the nuclear membrane as dying PLM touch neurons in a gain of function *mec-4* mutant (Fig. 1c).

### The role of intracellular calcium homeostasis

**Fig. 2.**
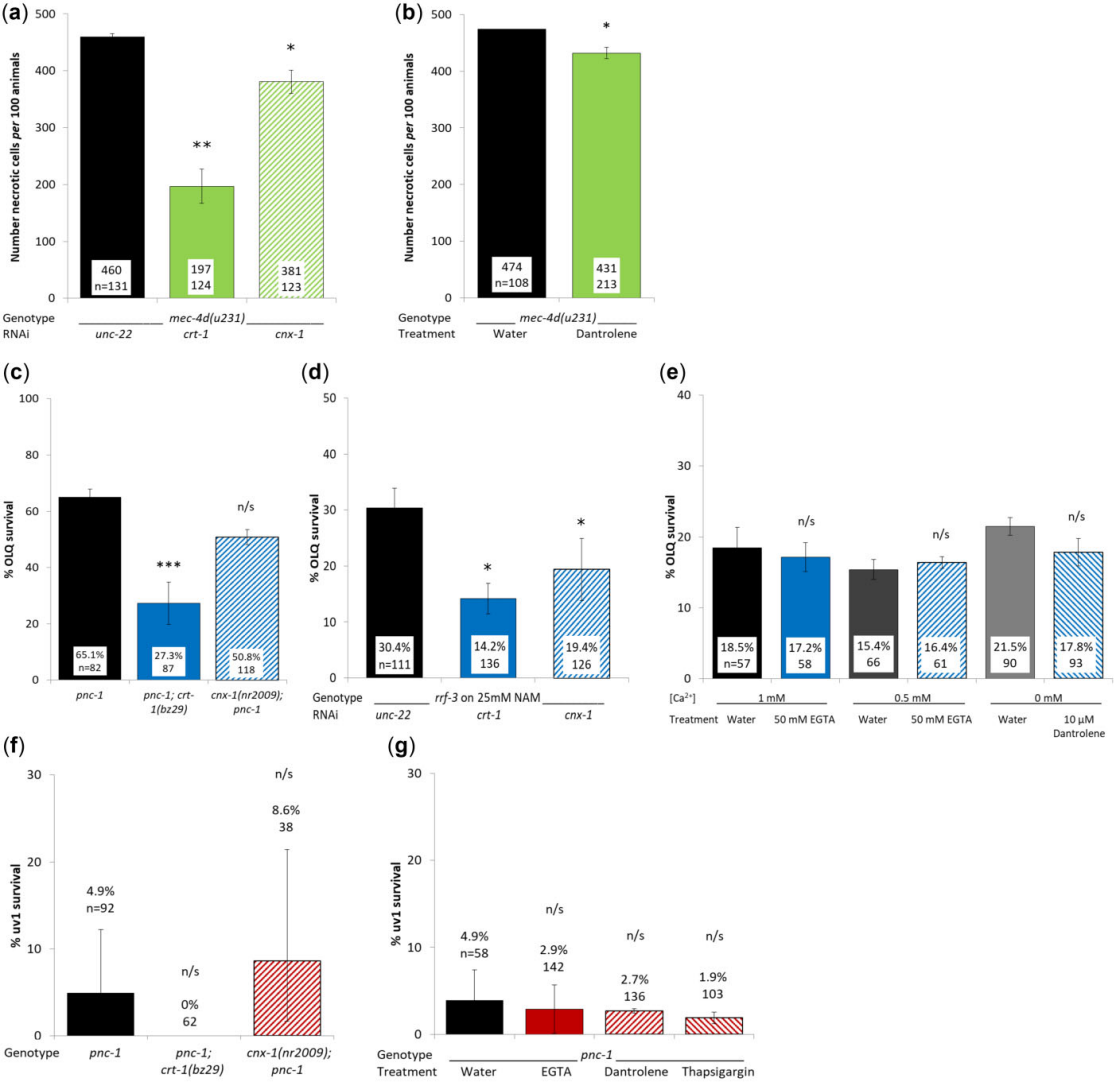
A calreticulin null mutation has opposing effects on OLQ and touch neuron survival. Calreticulin and calnexin RNAi knockdown (a) and exogenous dantrolene (b) increase touch cell survival in a *mec-4d(u231)* background. A calreticulin null mutation significantly reduces OLQ survival in a *pnc-1* background (c), as does both calreticulin and calnexin RNAi in RNAi sensitive worms on 25 mM NAM (d), but manipulation of Ca^2+^ levels has no effect (e). Neither calcium mutants nor modification of intracellular Ca^2+^ levels have any effect on uv1 survival (f,g). Percent survival and number of animals assayed are shown for each condition, with the exception of (a) and (b) where the number of necrotic animals *per* 100 animals is shown. a,b,d, and e) are means of 3 biological replicates and (g) is the mean of 2. Panels (c) and (f) are means of pooled data for each genetic background. Error bars are 1 s.e.m. for (a,b,d,e, and g), and 95% confidence intervals for (c and f). uv1 and OLQ survival for (c, e, f, and g) were scored using the psEx241 *ocr-4p::gfp* marker, OLQ survival for (d) was scored using the psEx279 *ocr-4p::gfp* marker and touch cell survival was scored using a tdIs5 *pmec-4::gfp* marker. ***, **, * and n/s represent *P* < 0.0001, *P* < 0.01, *P* < 0.05, and nonsignificant, respectively. Data were analyzed using Student’s *T*-test for (a and b) and the pairwise.prop.test in R with Holm *P*-value adjustment for (c–g).

Calcium homeostasis was shown to be important in *mec-4d* touch cell necrosis (Xu *et al.* 2001). Calreticulin (*crt-1*), an ER localized Ca^2+^ binding protein (Michalak *et al.* 1999) and calnexin (*cnx-1*), an important protein in Ca^2+^ homeostasis, are required for *mec-4d* and Ca^2+^-permeable ion channel *clhm-1* induced necrosis (Xu *et al.* 2001; Tanis *et al.* 2013). We found that *crt-1* and *cnx-1* RNAi knockdown significantly increased touch neuron survival in a *mec-4d(u2311)* background (Fig. 2a). It is also possible to manipulate intracellular calcium levels pharmacologically. Cytoplasmic Ca^2+^ levels can be reduced with a calcium chelator, such as ethylene glycol tetraacetic acid (EGTA), or by blocking Ca^2+^ release from ER with a ryanodine receptor antagonist, such as dantrolene (Zhao *et al.* 2001). Conversely cytoplasmic Ca^2+^ levels can be increased by blocking the activity of SERCA with thapsigargin, preventing uptake of Ca^2+^ from the cytoplasm into the ER (Thastrup *et al.* 1990). We found that exogenous dantrolene significantly reduced the number of necrotic touch cells *per* 100 animals (Fig. 2b).

However, a *crt-1(bz29)* allele had the opposite effect on OLQ survival, significantly reducing survival in a *pnc-1(pk9605); crt-1(bz29)* double mutant compared with *pnc-1(pk9605)* alone (Fig. 2c). To test whether the absence of effect of the calnexin allele on OLQ survival was due to the nature of the allele or the role of calnexin, we carried out RNAi knockdown of calreticulin and calnexin in an RNAi sensitive background supplemented with nicotinamide (NAM), a pharmacological equivalent to a *pnc-1* loss of function allele (Vrablik *et al.* 2009; Upadhyay *et al.* 2016). We found that calreticulin or calnexin RNAi produced a significant reduction of OLQ survival (Fig. 2d), but manipulation of calcium levels by supplementation of the media with EGTA or dantrolene had no effect (Fig. 2e). The difference in OLQ survival between *pnc-1* and *rrf-3* backgrounds may be due to either the properties of the *ocr-4::gfp* extrachromosomal array used or a difference in effective NAM concentration between *pnc-1* animals and *rrf-3* animals on 25 mM NAM. When we carried out the same experiments to assay uv1 survival, we found that none of the alleles or treatments had any effect (Fig. 2, f and g).

### Calpain and aspartic proteases promote uv1 but not OLQ necrosis

**Fig. 3.**
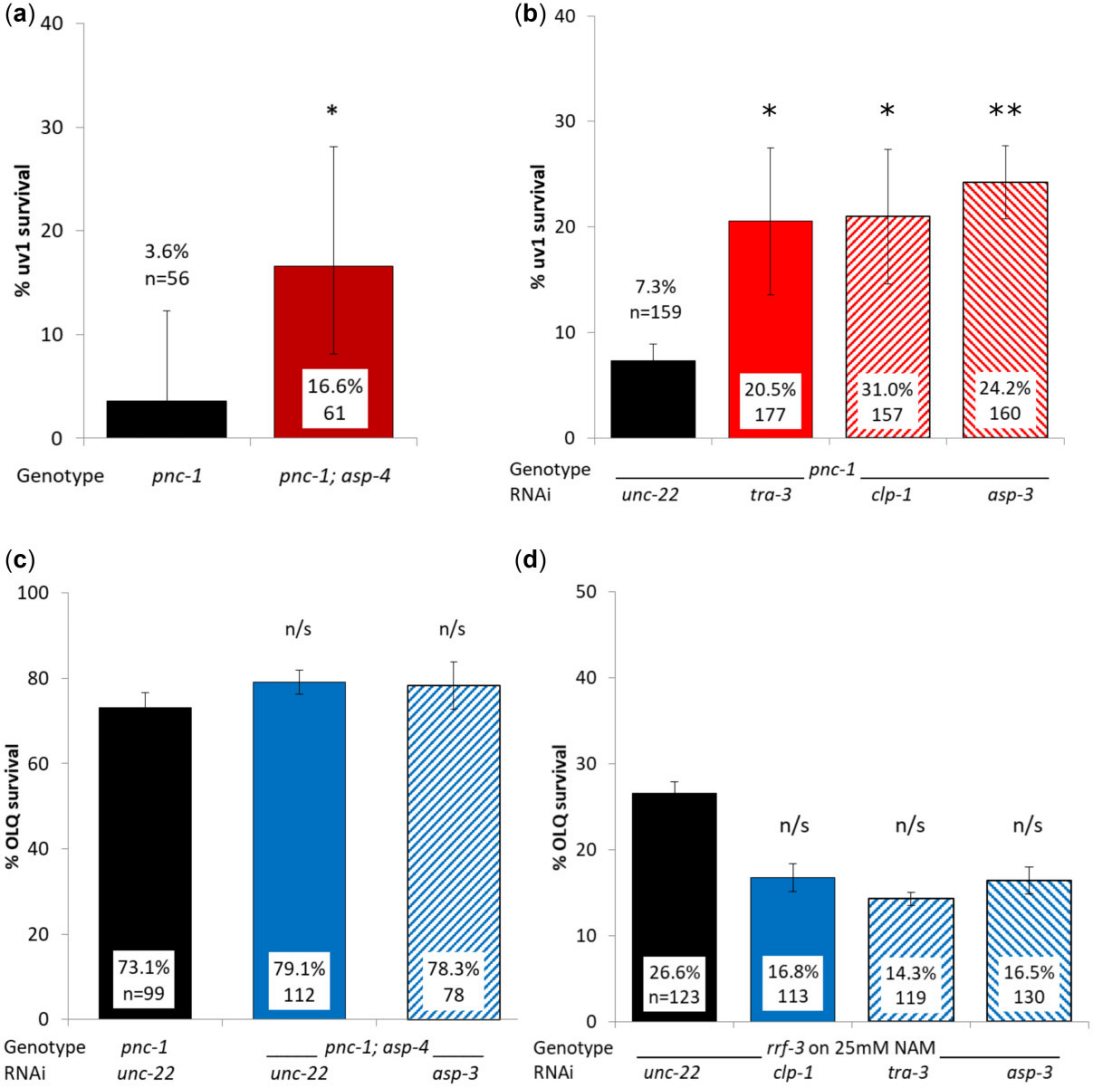
Reduction of calpain and aspartic protease function mutation promotes uv1 survival but does not significantly affect OLQ cell survival in a *pnc-1(pk9605)* mutant. An *asp-4(ok2693)* allele and RNAi of *tra-3*, *clp-1*, and *asp-3* increase uv1 survival a and b), but RNAi knockdown of the same genes has no effect on OLQ survival (c and d). Percent survival and number of animals assayed are shown for each condition. Panel (a) shows the means of pooled data for each genetic background; (b) the means for 4 biological replicates and (c and d) the means of three. Error bars are 95% confidence intervals for a and 1 s.e.m. for (b–d). uv1 and OLQ survival for (a–c) were scored using the psEx241 *ocr-4p::gfp* marker, OLQ survival for (d) was scored using the psEx279 *ocr-4p::gfp* marker. *, ** and n/s represent *P* < 0.01, *P* < 0.001, and nonsignificant, respectively, using pairwise.prop.test in R with the Holm *P*-value adjustment.

The calpain proteases *clp-1* and *tra-3* and the aspartic proteases *asp-3* and *asp-4* are required for *mec-4d* induced touch cell necrosis (Syntichaki *et al.* 2002). Previous work has shown that they are also required for uv1 necrosis as RNAi knockdown of *clp-1*, *asp-3*, and *asp-4* in a *pnc-1(ku212)* background increased uv1 survival (Huang and Hanna-Rose 2006). Both *pnc-1(ku212)* and *pnc-1(pk9605)* alleles are loss of function (Huang and Hanna-Rose 2006; Vrablik *et al.* 2009). We found that uv1 survival increased in a *pnc-1(pk9605); asp-4(ok2693)* double mutant compared with *pnc-1(pk9605)* alone (Fig. 3a), and RNAi knockdown of *asp-3, clp-1* or *tra-3* increased uv1 survival in a *pnc-1(pk9605)* background (Fig. 3b). We found no difference in OLQ survival when we compared either a *pnc-1(pk9605); asp-4(ok2693)* double mutant on control RNAi or the same animals on *asp-3* RNAi with *pnc-1(pk9605)* on control RNAi (Fig. 3c). Similarly, RNAi knockdown of *asp-3, clp-1* or *tra-3* in RNAi sensitive animals grown on NAM had no effect on OLQ survival (Fig. 3d).

### The role of the pro-apoptosis pathway

**Fig.4.**
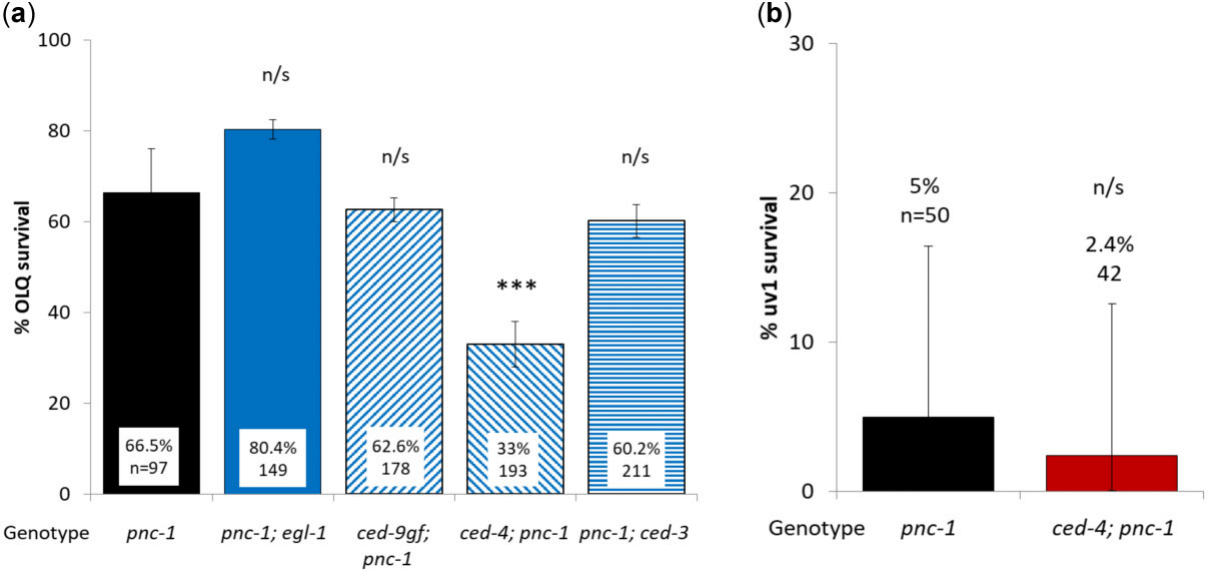
A knockout allele in the pro-apoptotic gene *ced-4*/*apaf1* reduces OLQ survival (a) but has no effect on uv1 survival (b). OLQ and uv1 survival was scored using an *ocr-4p::gfp* and *ida-1::gfp* marker, respectively. Mean % survival of pooled data for each genetic background and total number of animals assayed are shown for each condition. Error bars are 95% confidence intervals. *** and n/s represent *P* < 0.0001 and nonsignificant using pairwise.prop.test in R using the Holm *P*-value adjustment.

Dying OLQ and uv1 cells both show the characteristic expanded cytoplasm and degenerating nuclear membrane of cells dying by necrosis (Fig. 1, a and b) and do not have the classic apoptotic “button” morphology. Prior work showed that the *ced-4(n1162)* allele increased OLQ death (Upadhyay *et al.* 2016) but had no effect on uv1 survival (Huang and Hanna-Rose 2006). However, the possible role of other members of the pro-apoptotic pathway in OLQ or uv1 survival is unknown. We created double mutants with *pnc-1(pk9605)* and alleles of 4 members of the pro-apoptotic pathway: a loss of function allele in *egl-1(n1084n3082)*, the master regulator (Conradt and Horvitz 1998), a *ced-9(n1950)* gain of function allele in the negative regulator (Hengartner and Horvitz 1994) and loss of function alleles in the CED-3-activator *ced-4(n1162)* (Zou *et al.* 1997) and the executioner caspase *ced-3(n717)* (Xue *et al.* 1996). Based on the decrease in OLQ survival in a *ced-4(n1162); pnc-1(pk9605)* mutant we predicted that these alleles in other genes in the pathway would produce a similar effect. However, although we found that while the *ced-4(n1162)* mutant significantly reduced OLQ survival (Fig. 4a), mutations in the other pro-apoptotic pathway genes had no effect. The *ced-4(n1162)* allele had no effect on uv1 survival (Fig. 4b).

### Autophagy and axon guidance genes are required for OLQ survival

**Fig. 5.**
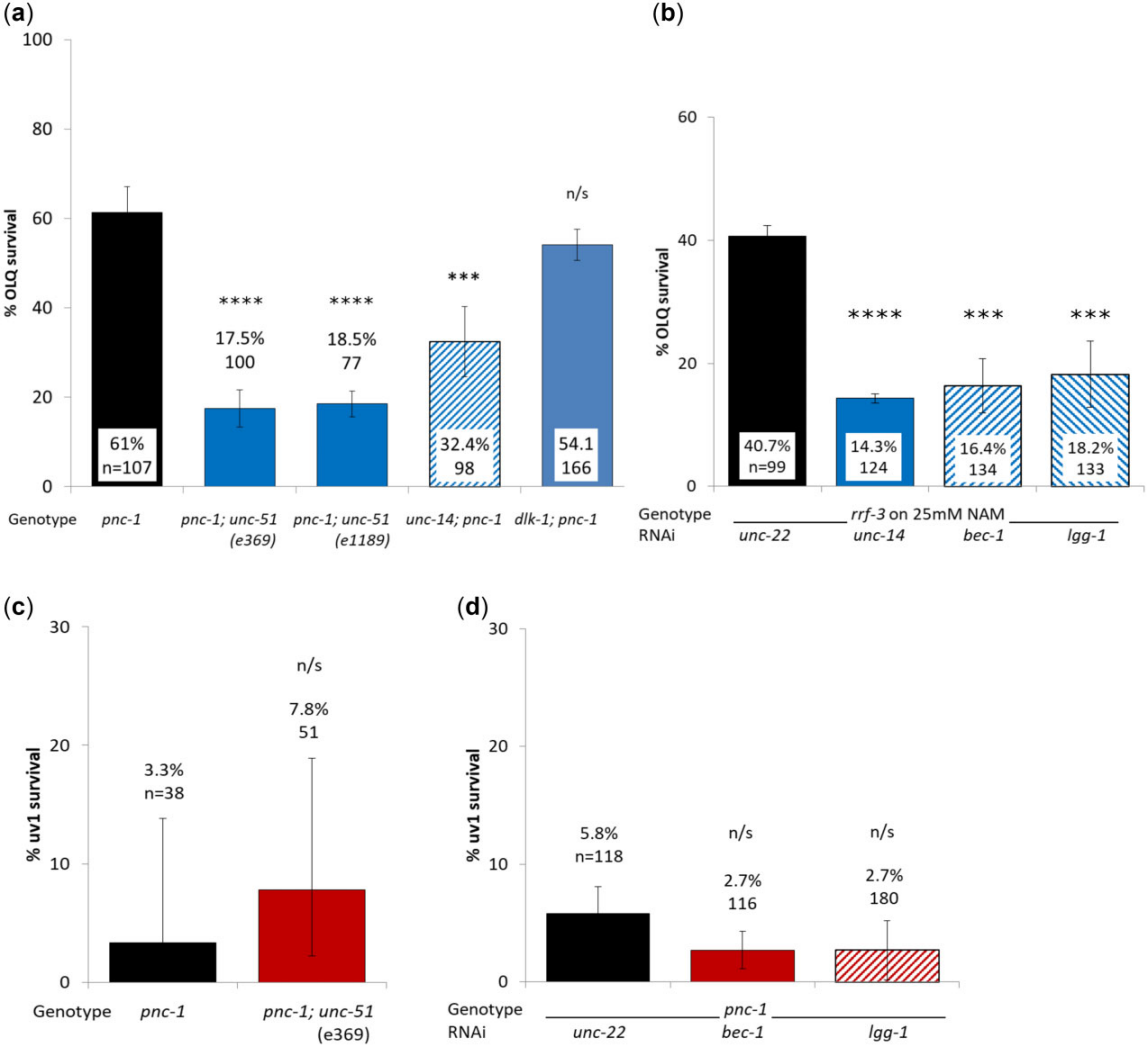
Genes involved in axon guidance and autophagy are required for OLQ, but not uv1 survival. Two different *unc-51* null alleles and an *unc-14* null mutation significantly reduced OLQ survival, but a *dlk-1* knockout did not (a). RNAi knockdown of 2 autophagy effector genes on NAM in an RNAi sensitive background significantly reduced OLQ survival (b). An *unc-51* null mutation (c) and RNAi of *bec-1* and *lgg-1* (d) had no effect on uv1 survival. OLQ survival was scored using the psEx241 *ocr-4p::gfp* marker for A and the psEx279 *ocr-4p::gfp* marker for (b). uv1 survival was scored using an *ida-1::gfp* marker. Percent survival and number of animals assayed are shown for each condition. Panels (a) and (c) show the means of pooled data for each genetic background; (b) and (d) the means for 3 and 2 biological replicates, respectively. Error bars are 95% confidence intervals for (a and c), and 1 s.e.m. for (b and d). ***, ****, and n/s represent *P* < 0.001, *P* < 0.001, and nonsignificant using pairwise.prop.test in R using the Holm *P*-value adjustment.

Finally, another form of cell death is autophagy or “self-eating” in response to nutrient deficient conditions (Takeshige *et al.* 1992) and is required for *mec-4d-*induced touch cell necrosis (Samara *et al.* 2008). In *C. elegans* the master autophagy regulator is the serine-threonine kinase *unc-51*, an ortholog of Atg1/ULK2 (Ogura *et al.* 1994) that is also important in axon guidance (Ogura *et al.* 1994; Lai and Garriga 2004; Ogura and Goshima 2006), cell size regulation (Aladzsity *et al.* 2007) and dauer development (Meléndez *et al.* 2003). If OLQ or uv1 cells were dying at least in part by autophagy, then an *unc-51* mutant would increase survival. Instead, we found that both *unc-51(e369)* and *unc-51(e1189)* alleles significantly reduced OLQ survival in a *pnc-1(pk9605)* background (Fig. 5a), as did RNAi knockdown of downstream autophagy genes involved in autophagy vesicle nucleation (*bec-1*, Meléndez *et al.* 2003) or vesicle expansion (*lgg-1*, Kirisako *et al.* 2000) (Fig. 5b). Neither an *unc-51* loss of function allele or RNAi of *bec-1* or *lgg-1* had any effect on uv1 survival (Fig. 5, c and d).

UNC-51 interacts with UNC-14 in its axon guidance role (Ogura *et al.* 1997; Lai and Garriga 2004). If this interaction was critical to OLQ survival, then an *unc-14* loss of function allele would have the same effect on OLQ survival as loss of UNC-51 activity. We found that reducing UNC-14 function did significantly reduce OLQ survival (Fig. 5, a and b), although to a lesser extent than either of the *unc-51* mutants. In *C.* *elegans*, neuron generation and neuroregeneration is controlled by DLK-1, a Dual Leucine-zipper Kinase homolog (Ghosh-Roy *et al.* 2010), which requires LIN-14 signaling and autophagy (Ko *et al.* 2020). However, we found no difference in OLQ survival between a *dlk-1(ju476); pnc-1(pk9605)* double mutant and *pnc-1(pk9605)* alone (Fig. 5a).

### *pnc-1* animals are partially nose touch insensitive on poor quality food

OLQ neurons are mechanosensory cells that, together with the ASH and FLP neurons, mediate the gentle nose touch “stop and reverse” response to objects blocking the animals forward motion (Kaplan and Horvitz 1993). We have previously shown that *pnc-1(pk9605)* animals and wild-type animals grown on NAM supplemented media have a reduced nose touch response (Upadhyay *et al.* 2016). We also know that growth of *pnc-1(pk9605)* animals on UV-killed *E. coli* exacerbates certain *pnc-1* phenotypes and supplementation with 1 mM NAD^+^ can restore function to certain tissues in *pnc-1* mutants grown on UV-killed *E. coli* (Vrablik *et al.* 2009; 2011). To investigate the requirement for live food and NAD^+^ for OLQ function, we compared the number of times *pnc-1* L4s stopped when they moved into a human eye lash with that of wild-type animals and *glr-1* mutants, which have a drastically reduced light nose touch response (Hart *et al.* 1995), and how that response varied on UV-killed *E. coli* and UV-killed *E. coli* supplemented with 1 mM NAD^+^.

**Fig. 6.**
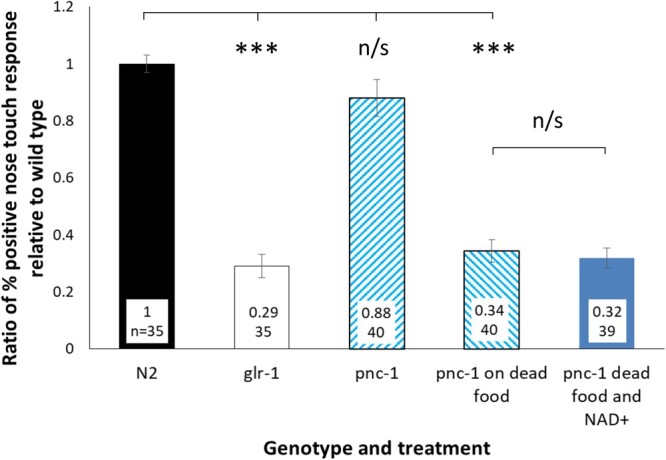
pnc-1 L4 animals show a defective response to gentle nose touch when grown on UV-killed *E. coli* which is not rescued by supplementation with 1 mM NAD^+^. N2 and *glr-1(n2461)* animals grown on live *E. coli* were used as controls (Hart *et al.* 1995). Number of animals assayed and mean percentage of animals from 4 biological repeats that responded to nose touch relative to wild type are shown. Error bars are 1 s.e.m. Percentages were analyzed by *T*-test. *** and n/s represent *P*-values of <0.0001 and nonsignificant, respectively.

We found that *pnc-1* animals are not significantly less sensitive to gentle nose touch than wild-type animals (Fig. 6). However, when *pnc-1* animals were grown on UV-killed *E. coli* significantly fewer animals stopped on gentle nose touch compared with wild-type animals, with a response similar to that of touch insensitive *glr-1* animals (Fig. 6). Interestingly, this reduced nose touch sensitivity on dead *E. coli* was not affected by supplementation with 1 mM NAD^+^.

## Discussion

We have shown, both in this and prior work (Huang and Hanna-Rose 2006; Upadhyay *et al.* 2016) that mechanosensory OLQ neurons and uv1 neuroendocrine cells die in a *pnc-1* background as a result of excess nicotinamide. Dying OLQ and uv1 cells present the same stereotyped morphological changes, namely an increasingly vacuolar and expanding cytoplasm and disintegrating nuclear membrane, as seen in *mec-4d* and degenerin induced touch cell necrosis (Driscoll and Chalfie 1991; Hall *et al.* 1997). So based on morphological characteristics alone, OLQ and uv1 cells die by necrosis. However, there many different forms of necrosis (Fricker *et al.* 2018). We sought to determine by which of the known mechanisms of cell death OLQ and uv1 cells die.

One of the key molecular characteristics of several forms of necrosis is the rise in intracellular calcium levels (Xu *et al.* 2001; Yamashima 2004; Fricker *et al.* 2018). We replicated prior work showing that calreticulin and calnexin function is required for touch cell necrosis, but we found that the level of protection provided by both *crt-1* and *cnx-1* knockdown to be lower than previously published (Xu *et al.* 2001). Similarly, we saw a small increase in touch cell survival in animals grown in the presence of dantrolene, which blocks calcium release from the ER *via* the ryanodine receptor. When we repeated these experiments in a *pnc-1* mutant to investigate their effect on uv1 and OLQ necrosis we found very different results. uv1 survival was not affected by either *crt-1* or *cnx-1* loss of function or the reduced availability of intracellular calcium, similar to that of *deg-3*-induced touch cell necrosis (Xu *et al.* 2001) or MPP+ induced dopaminergic neurodegeneration (Pu and Le 2008), suggesting that overactivation of the OCR-4/OSM-9 TRPV channel in uv1 cells does not trigger a change in intracellular calcium levels. In contrast, loss of calreticulin and calnexin function significantly decreased OLQ survival. The requirement of calreticulin and calnexin function for OLQ survival may be tied to their other functions in stress response (Park *et al.* 2001) or lipid homeostasis (Wang *et al.* 2017) rather than their role in calcium homeostasis, as pharmacological manipulation of intracellular calcium levels had no effect on OLQ survival.

Downstream of an increase in calcium levels is the activation of calpain and aspartic proteases (Syntichaki *et al.* 2002). We were able to replicate prior results showing that uv1 necrosis requires ASP-3, ASP-4, CLP-1, and TRA-3 function (Huang and Hanna-Rose 2006). Together with prior work these results strongly suggest a role for calpains and aspartic proteases in uv1 necrosis. In contrast, OLQ survival was not affected by either the same *asp-4(ok2693)* allele that promoted uv1 survival or by *asp-3*, *clp-1*, or *tra-3* RNAi, even though both uv1 and OLQ cells die from the same insult (Upadhyay *et al.* 2016). It is possible that one or more of the 17 other aspartic protease genes in the *C. elegans* genome (Kim *et al.* 2018) are required for OLQ necrosis, such as *asp-1* in *Bacillus thuringiensis* Cry6A induced necrosis (Zhang *et al.* 2016). It is also possible that neither calpain nor aspartic proteases are required at all, such as in linker cell death (Abraham *et al.* 2007).

We have already shown that *ced-4* is required for OLQ survival (Upadhyay *et al.* 2016) and is not needed for uv1 necrosis or survival (Huang and Hanna-Rose 2006), so we asked if the rest of the pro-apoptotic pathway was required for OLQ survival. We found that although *ced-4* was required for OLQ survival none of the other apoptosis genes tested were. A *ced-9* gain of function allele, which reduces CED-4 activity (Hengartner and Horvitz 1994), does not have any effect suggesting that the role of CED-4 in OLQ survival is independent of CED-9 activity. *ced-3* and *ced-4* are required for axon outgrowth after axotomy (Pinan-Lucarre *et al.* 2012), but *ced-3* was not required for OLQ survival. It may be that the *ced-4* pro-survival role in OLQ is related to its dual role in apoptosis, where the long form *ced-4L* transcript is pro-survival (Shaham and Horvitz 1996), but that doesn’t explain why the *ced-9* gain of function mutation, which inhibits the *ced-4L* isoform (Shaham and Horvitz 1996), does not reduce OLQ survival or why *ced-4* is not required for uv1 survival.

Autophagy is a response to nutrient limitation and protein aggregation. As such it can be pro-survival by repurposing cellular components for other purposes or reducing toxic protein load, but if it proceeds too far can also lead to cell death, a conundrum dubbed Janus-faced autophagy (Vellai *et al.* 2007). Although autophagy is required for touch cell necrosis (Toth *et al.* 2007; Samara *et al.* 2008) the master autophagy regulator *unc-51* and the effector genes *bec-1* and *lgg-1* were instead required for OLQ survival and played no role in uv1 necrosis or survival. Autophagy is neuroprotective in mice (Hara *et al.* 2006; Komatsu *et al.* 2006) and promotes cell survival in *Pseudomonas auruginosa* infections (Zou *et al.* 2014) as well as being required for axon regrowth in PLM neurons after injury (Ko *et al.* 2020). UNC-51 also plays a major role in axon elongation (Ogura *et al.* 1994) and interacts with UNC-14 (Lai and Garriga 2004). We found that UNC-14 function was also required for OLQ survival, but the *dlk-1* pathway that controls neuroregeneration (Hammarlund *et al.* 2009) and axon regrowth (Ko *et al.* 2020) is not. Our results suggest that both autophagy and some aspect of the control of neuron formation or axon elongation is required for OLQ survival when NAM levels are high.

Previous work showed that *pnc-1* mutants have a reduced nose touch response and a foraging defect, most likely due to prolonged TRPV channel signaling by its NAM agonist (Upadhyay *et al.* 2016). Nose touch response in our assay was not significantly reduced in a *pnc-1* mutant on live food in contrast with previous work (Upadhyay *et al.* 2016), perhaps due to differences in the assay where we allowed the animals to move into the eyelash compared with Upadhyay *et al.* who touched the animal with the eyelash. However, growing *pnc-1* animals on dead *E. coli*, which are unable to compensate for the reduced production of NAD^+^ (Vrablik *et al.* 2009), reduced their nose touch response to a level similar to *glr-1* mutants. Growth on dead food increases the severity of the *pnc-1* gonad developmental delay and male spicule phenotypes (Vrablik *et al.* 2009; 2011) and supplementing dead food with NAD^+^ reverses the effect of dead food on these phenotypes, but supplementary NAD^+^ did not restore nose touch in worms on dead food. There may be a separate process that modulates OLQ survival which relies on a different NAD^+^ salvage pathway metabolite, such as nicotinic acid, that is absent in UV-killed *E. coli.* Or the ASH or FLP neurons, which contribute the majority of function in gentle nose touch response (Kaplan and Horvitz 1993), are sensitive to reduced levels of NAD^+^ or salvage pathway intermediates but this sensitivity cannot be compensated for by exogenous NAD^+^.

Our work has shown that there are at least 3 different types of necrosis in *C. elegans*. The canonical *mec-4d*-induced touch cell necrosis, which is calcium dependent (Xu *et al.* 2001) and relies on calpain and aspartic protease function (Syntichaki *et al.* 2002), with a role for autophagy (Toth *et al.* 2007; Samara *et al.* 2008). *acr-2*-induced cholinergic neurodegeneration (Barbagallo *et al.* 2010) and 6-OHDA or *trp-4*-induced dopaminergic neurodegeneration (Nagarajan *et al.* 2014; Offenburger *et al.* 2018) also fit this model, at least in part. Then there is NAM-induced uv1 necrosis, which is also calpain and aspartic protease dependent, but does not require elevated intracellular calcium levels or functional autophagy. Finally, we have NAM-induced OLQ necrosis, which is prevented by CRT-1, CED-4, UNC-51, BEC-1, LGG-1, and UNC-14 function. As OLQ neurons degenerate slowly over developmental time, with axon and dendrite blebbing occurring before cell swelling (Upadhyay *et al.* 2016), these proteins may play a neuroprotective role, perhaps reprising their role in axon outgrowth (Lai and Garriga 2004) and regeneration (Pinan-Lucarre *et al.* 2012).

Why are the mechanisms underpinning NAM-induced uv1 and OLQ necrosis different? One possible explanation is that OLQ cells start dying in late L2/early L3 (data not shown), over 20 h after their specification during embryogenesis (Sulston *et al.* 1983), whereas uv1 cells die within a few hours of being born in the L4 stage (Huang and Hanna-Rose 2006). Another is that OLQ neurons are less sensitive to NAM, requiring higher NAM concentrations and more time to achieve the same degree of penetrance (Upadhyay *et al.* 2016). Finally, OLQ cells are mechanosensory neurons (Kaplan and Horvitz 1993) whereas uv1 cells are tyramine synthesizing neuroendocrine cells (Alkema *et al.* 2005). We do not know which of the above is responsible, if any, for the differences between uv1 and OLQ necrosis, though time to die and NAM sensitivity are almost certainly linked.

We have shown that necrosis in *C. elegans* is not a monolithic type of cell death, even when the cells die from the same insult. We have shown that neither elevated calcium levels nor calpain and aspartic protease activity are universally required. In addition, our study of OLQ necrosis has also highlighted other possible mechanisms of cell survival, requiring calreticulin, calnexin, CED-4, and key players in axon guidance, all of which deserve further study.

## Data availability

Strains and plasmids are available upon request. The authors affirm that all data necessary for confirming the conclusions of the article are present within the article, figures, and tables.
